# Transciptomic and histological analysis of hepatopancreas, muscle and gill tissues of oriental river prawn (*Macrobrachium nipponense*) in response to chronic hypoxia

**DOI:** 10.1186/s12864-015-1701-3

**Published:** 2015-07-03

**Authors:** Shengming Sun, Fujun Xuan, Hongtuo Fu, Jian Zhu, Xianping Ge, Zhimin Gu

**Affiliations:** Key Laboratory of Genetic Breeding and Aquaculture Biology of Freshwater Fishes, Ministry of Agriculture, Freshwater Fisheries Research Center, Chinese Academy of Fishery Sciences, Wuxi, 214081 People’s Republic of China; Jiangsu Provincial Key Laboratory of Coastal Wetland Bioresources and Environmental Protection, Yancheng City, Jiangsu Province 224002 People’s Republic of China; Agriculture Ministry Key Laboratory of Healthy Freshwater Aquaculture, Zhejiang Institute of Freshwater Fisheries, Huzhou, 313001 People’s Republic of China

**Keywords:** *Macrobrachium nipponense*, RNA-Seq, Gene expression, Hypoxia

## Abstract

**Background:**

Oriental river prawn, *Macrobrachium nipponense*, is a commercially important species found in brackish and fresh waters throughout China. Chronic hypoxia is a major physiological challenge for prawns in culture, and the hepatopancreas, muscle and gill tissues play important roles in adaptive processes. However, the effects of dissolved oxygen availability on gene expression and physiological functions of those tissues of prawns are unknown. Adaptation to hypoxia is a complex process, to help us understand stress-sensing mechanism and ultimately permit selection for hypoxia- tolerant prawns, we performed transcriptomic analysis of juvenile *M. nipponense* hepatopancreas, gill and muscle tissues by RNA-Seq.

**Results:**

Approximately 46,472,741; 52,773,612 and 58,195,908 raw sequence reads were generated from hepatopancreas, muscle and gill tissues, respectively. A total of 62,722 unigenes were generated, of the assembled unigenes, we identified 8,892 genes that were significantly up-regulated, while 5,760 genes were significantly down-regulated in response to chronic hypoxia. Genes from well known functional categories and signaling pathways associated with stress responses and adaptation to extreme environments were significantly enriched, including genes in the functional categories “response to stimulus”, “transferase activity” and “oxidoreductase activity”, and the signaling pathways “oxidative phosphorylation”, “glycolysis/gluconeogenesis” and “MAPK signaling”. The expression patterns of 18 DEGs involved in hypoxic regulation of *M. nipponense* were validated by quantitative real-time reverse-transcriptase polymerase chain reactions (qRT-PCR; average correlation coefficient = 0.94). In addition, the hepatopancreas and gills exhibited histological differences between hypoxia and normoxia groups. These structural alterations could affect the vital physiological functions of prawns in response to chronic hypoxia, which could adversely affect growth and survival of *M. nipponense*.

**Conclusions:**

Gene expression changes in tissues from the oriental river prawn provide a preliminary basis to better understand the molecular responses of *M. nipponense* to chronic hypoxia. The differentially expressed genes (DEGs) identified in *M. nipponense* under hypoxia stress may be important for future genetic improvement of cultivated prawns or other crustaceans through transgenic approaches aimed at increasing hypoxia tolerance.

**Electronic supplementary material:**

The online version of this article (doi:10.1186/s12864-015-1701-3) contains supplementary material, which is available to authorized users.

## Background

In aquatic environments, oxygen levels often fluctuate and crustaceans experience hypoxic conditions on a regular basis [1]. *Macrobrachium nipponense*, which is widely distributed in freshwater and low-salinity regions of estuaries, is one of the most important aquaculture species in China [2]. This freshwater prawn is an attractive model to study responses to hypoxia because it is relatively susceptible to hypoxic conditions compared to other crustaceans [3]. Hypoxic conditions cause stress that can inhibit the optimal development of crustaceans, resulting in reduced frequency of molts, metabolic changes, avoidance behavior, slow growth, suppression of immune function and even death [4–9]; however, few genetic markers to evaluate population genetics and perform phylogenic analysis [10–12] has meant that the molecular basis of chronic hypoxia exposure in *Macrobrachium* spp. is not well characterized. Recently, high-throughput transcriptomic sequencing was performed on the Illumina platform and this provided a genomic basis for further investigation of the hypoxia adaptation mechanisms of *M. nipponense* [13]. Breeding programs also eagerly desire a better understanding of physiological and genetic mechanisms underlying sensitivity to oxygen limitation.

Comparative studies between organisms inhabiting distinct environments can provide insight into the mechanisms that underlie responses to environmental differences [14]. In some cases, artificial treatments are used to create these differences in conditions and to facilitate comparisons [15–17]. To better understand the physiological and genetic changes in *M. nipponense* in response to hypoxia, comparative analysis between normoxic and hypoxic conditions is necessary. Previous research in *M. nipponense* has focused on the effects of hypoxia on respiratory metabolism [18], and there is growing evidence that changes in molecular indicators, such as energy production, antioxidant enzymes and oxygen-carrying proteins, can effectively imply chronic and acute hypoxia exposure in invertebrates [19–23]. When facing hypoxia, one of the main challenges of any tissue is to maintain energy levels either by increasing anaerobic energy-production, improving energy-utilization or by lowering energy consumption; however, cellular adaptations to chronic hypoxia in hepatopancreas, gill and muscle tissues in prawns remain unexplained. As a result of this knowledge gap, large-scale expressed sequence tag (EST) libraries of *M. nipponense* have been sequenced [24–26], as these should allow more comprehensive molecular studies. Recently, high-throughput RNA-sequencing (RNA-Seq) has emerged as a strategy for analyzing the functional complexity of transcriptomes [13, 27, 28]. With the emergence of next generation sequencing, RNA-Seq is a relatively new technology allowing transcriptomic studies of the whole genome. Compared to traditional cDNA microarrays, RNA-Seq provides deep sequencing data for direct quantification of transcripts, which makes it more sensitive for detecting all expressed genes without the need for EST collection, probe synthesis, microarray design and hybridization.

In this present study, we used RNA-Seq to investigate genome-wide gene expression differences in *M. nipponense* in response to chronic hypoxia. To provide insight into the mechanisms underlying hypoxia-sensitivity in *M. nipponense*, it is necessary to identify hypoxia-related genes such as those in the hepatopancreas, muscle and gill tissues, as these are the most important organs for metabolism of nutrients, energy storage and immune responses, respectively. Our study highlights the pathways that react in response to chronic hypoxia through gene ontology (GO) and pathway analyses. This present study provides useful information to better understand the genetic-level responses of *M. nipponense* when exposed to chronic hypoxia.

## Results and discussion

### RNA-Seq data processing, reference assembly and alignment

**Fig. 1:**
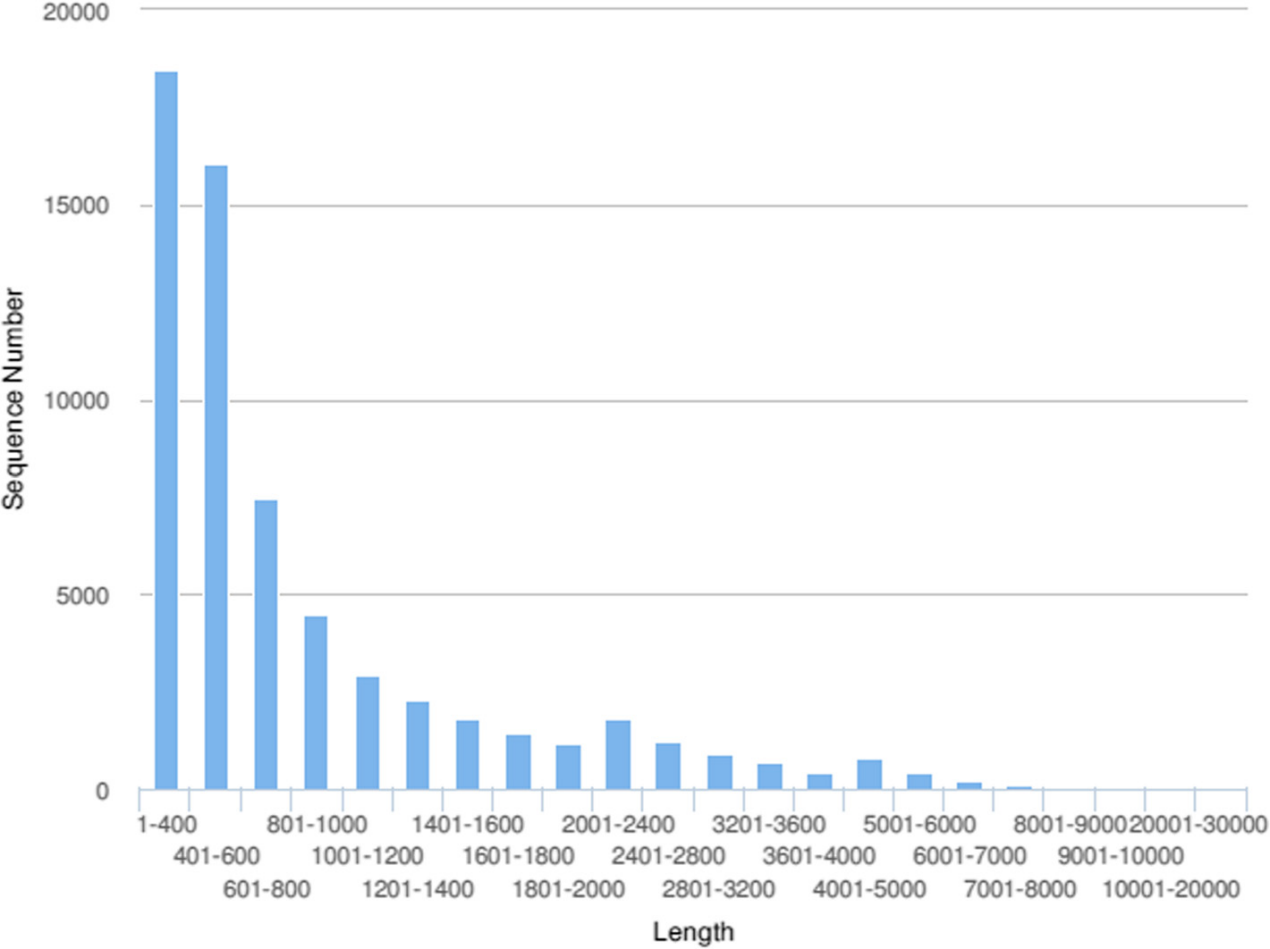
Length distribution of assembled transcriptome unigenes from *Macrobrachium nipponense*. The x-axis indicates contig size and the y-axis indicates the number of unigenes of each size

To provide comprehensive understanding of expression differences between *M. nipponense* cultured in normoxic and hypoxic conditions, we collected and deep sequenced RNA samples from hepatopancreas, muscle and gill tissues. A total of 308,348,065 paired-end reads were generated from six samples with 101-bp read length. The number of sequences in each sample ranged from 45.6 to 58.2 million (Table 1). After removal of ambiguous nucleotides, low-quality sequences (Phred quality scores <20), contaminated microbial sequences and ribosomal RNA sequences, a total of 265,294,865 cleaned reads (86 %) were harvested for further analysis. The cleaned sequences in each sample ranged from 31.5 to 52.7 million reads, thus confirming the stability and consistency of sampling, library preparation and sequencing methodologies. Using the Trinity assembly program, we generated a total of 62,722 unigenes, the average length was 960 bp, and the N50 length was 1,450 bp. (Table 2). The length distribution of unigenes is shown in Fig. 1. The cleaned reads of the six samples were pooled and assembled by the Trinity assembler to generate a reference transcriptome assoiated with published EST datas [29]. According to Gene Ontology (GO), an internationally standardized gene functional classification system, 11,104 non-redundant unigenes were classified into three major functional categories (biological process, cellular component and molecular function).

**Table 1:** Summary of sequence data generated for the *Macrobrachium nipponense* transcriptome, and quality filtering

Group	Tissue	Reads	Clean reads	Total Clean Nucleotides (nt)	Q20 percentage
Normoxia	Hepatopancreas	46,472,741	32,323,574	4,865,587,148	98.56 %
	Muscle	52,773,612	50,213,656	4,822,295,055	98.42 %
	Gill	58,195,908	52,684,676	4,741,620,840	98.26 %
Hypoxia	Hepatopancreas	45,590,728	31,457,005	4,731,627,788	98.35 %
	Muscle	49,727,598	47,295,194	4,712,350,852	98.15 %
	Gill	55,587,478	51,320,754	4,618,867,860	97.78 %

**Table 2:** Assembly statistics of reads

Parameter	Numbers
Number of Unigene	62,722
Total bases of Unigene (bp)	36,589,624
Unigene mean lengths (bp)	960
Minimum length (bp)	300
Max length (bp)	26,388
N50 length	1450

**Table 3:** GO enrichment analysis of genes up- or down-regulated in response to hypoxia stress

Tissue	GO term	Definition	No. up- or down- regulated	Total in category	*P*-value
Hepatopancreas	Up				
	GO:0016491	Oxidoreductase activity	58	773	8.20E-09
	GO:0003824	Catalytic activity	33	2720	4.50E-08
	GO:0055114	Oxidation-reduction process	62	551	5.21E-08
	Down				
	GO:0002376	Immune system process	57	1697	4.58E-08
	GO:0022904	Respiratory electron transport chain	47	119	8.09E-08
	GO:0043167	Ion binding	78	2466	5.43E-08
Muscle	Up				
	GO:0003824	Catalytic activity	104	2720	7.99E-08
	GO:0045182	Transcription regulator activity	113	2466	6.77E-06
	Down				
	GO:0006030	Chitin metabolic process	11	58	0.007
	GO:0006936	Muscle contraction	4	31	0.011
Gill	Up				
	GO:0016901	Oxidoreductase activity	32	523	0.004
	GO:0007154	Cell communication	28	152	2.68E-07
	Down				
	GO:0050896	Response to stimulus	15	373	8.66E-08
	GO:0042221	Response to chemical stimulus	30	251	3.81E-06
	GO:0002376	Immune system process	28	75	3.07E-07

**Table 4:** List of DEGs in hepatopancreas, muscle and gill tissues of pranws in response chronic hypoxia

Genes	Gene ID	Differential expression tissues		
caspase-3	isotig20204			gill
inhibitor of apoptosis	isotig00582	hepatopancreas	muscle	
extracellular copper/zinc superoxide dismutase	isotig04224	hepatopancreas	muscle	gill
glutathione peroxidase	isotig15120	hepatopancreas		gill
glutathione S-transferase	isotig17220	hepatopancreas	muscle	gill
peroxidase	isotig02186		muscle	
phosphoenolpyruvate carboxykinase	isotig03978		muscle	
hexokinase	isotig07588		muscle	
pyruvate kinase	isotig03788		muscle	
lactate dehydrogenase	isotig03261		muscle	
arginine kinase 2	isotig02307	hepatopancreas	muscle	
Na^+^/K^+^ ATPase	isotig02174			gill
cathepsin L	isotig35781	hepatopancreas	muscle	
tropomyosin-2	isotig00694		muscle	
tubulin	isotig05597		muscle	
myosin	isotig11489	hepatopancreas	muscle	
hemocyanin	isotig34775	hepatopancreas		
carbonic anhydrase I	isotig02566	hepatopancreas		
cytochrome c oxidase subunit I	isotig00782	hepatopancreas	muscle	gill
cytochrome oxidase subunit I	isotig05039	hepatopancreas		gill
NADH dehydrogenase subunit 1	isotig04226			gill
NADH dehydrogenase subunit 4	isotig02071	hepatopancreas		gill
ATP synthase	isotig00758	hepatopancreas		
heat shock protein 70	isotig27058		muscle	
heat shock protein 90	isotig04367	hepatopancreas	muscle	
heat shock protein 21	isotig12922		muscle	
heat shock protein 60	isotig03937			gill
fatty acid synthase	isotig08686			gill
prophenoloxidase	isotig00567			gill
serine proteinase-like protein	isotig03292		muscle	
plus agglutinin	isotig06982			gill
akirin	isotig02975		muscle	gill
C-type lectin	isotig11437	hepatopancreas	muscle	gill
antimicrobial peptide	isotig29146			gill
alpha-2-Macroglobulins	isotig00388			gill
β-1,3-glucan binding protein	isotig26537			gill

**Table 5:** KEGG Pathway enrichment analysis of differentially expressed genes in response to hypoxia stress (P-value <0.05)

KEGG Pathway	Hepatopancreas		Muscle		Gill	
	Number of genes	q-value	Number of genes	q-value	Number of genes	q-value
Oxidative phosphorylation	31	0.000	8	0.782	42	0.047
MAPK signaling pathway	10	0.000	9	0.006	9	0.028
Lysosome	26	0.014	8	0.306	36	0.056
Glycolysis / gluconeogenesis	28	0.000	12	0.006	15	0.183
Fatty acid metabolism	33	0.012	5	0.818	11	0.000
Citrate cycle (TCA cycle)	6	0.009	6	0.029	11	0.292
Starch and sucrose metabolism	17	0.000	3	0.194	13	0.048
Insulin signaling pathway	24	0.013	7	0.025	17	0.975
Peroxisome	24	0.000	2	0.000	12	0.045
p53 signaling pathway	4	0.602	7	0.013	9	0.032

### Identification of DEGs

**Fig. 2:**
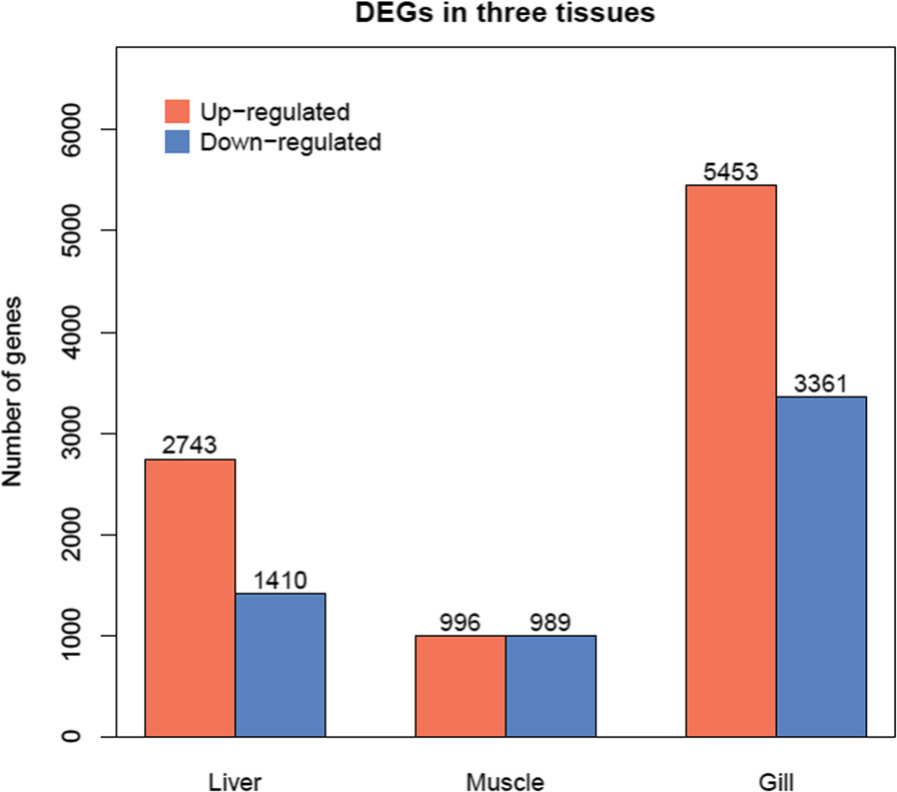
DEGs in three tissues from *Macrobrachium nipponense* between normoxia and hypoxia conditions

**Fig. 3:**
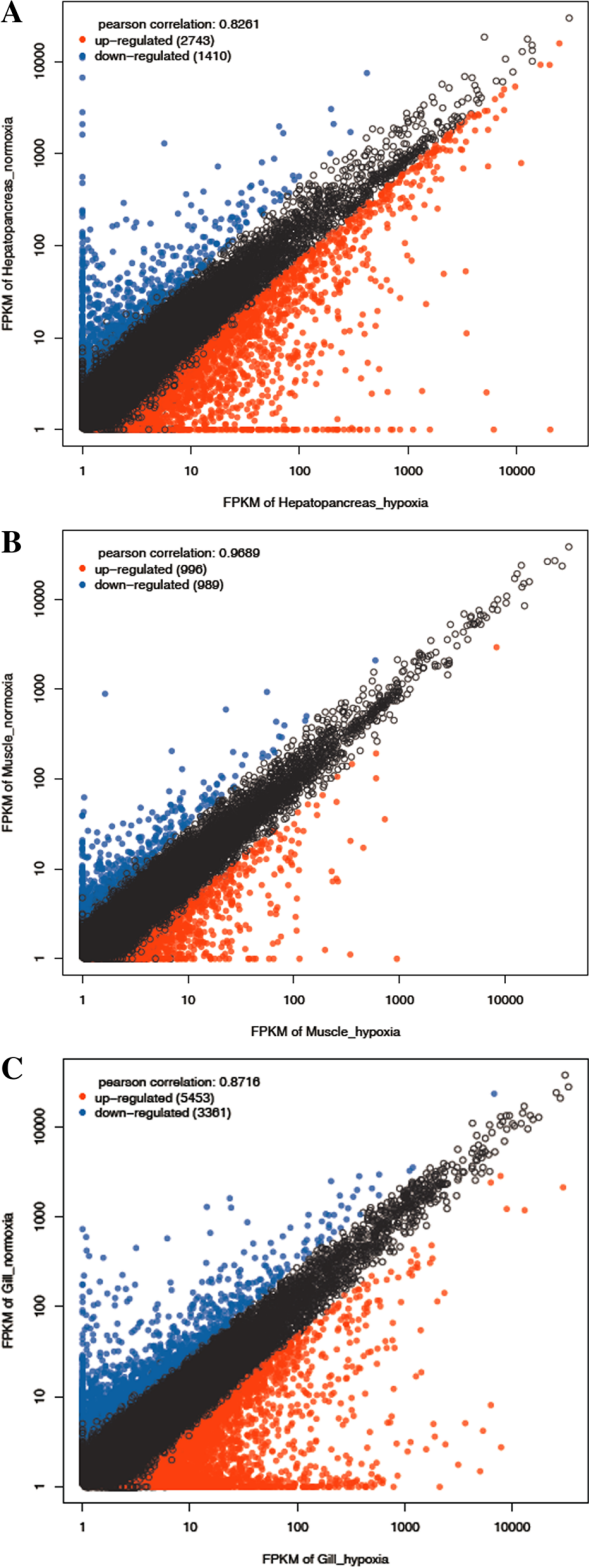
Scatter-plot showing gene expression in three tissues from *Macrobrachium nipponense*. Average FPKM values for each gene in the gill **a**, hepatopancreas **b** and muscle **c** tissues in hypoxia conditions correlated to average FPKM values for each gene in gill **a**, hepatopancreas **b** and muscle **c** tissues in normoxia conditions

**Fig. 4:**
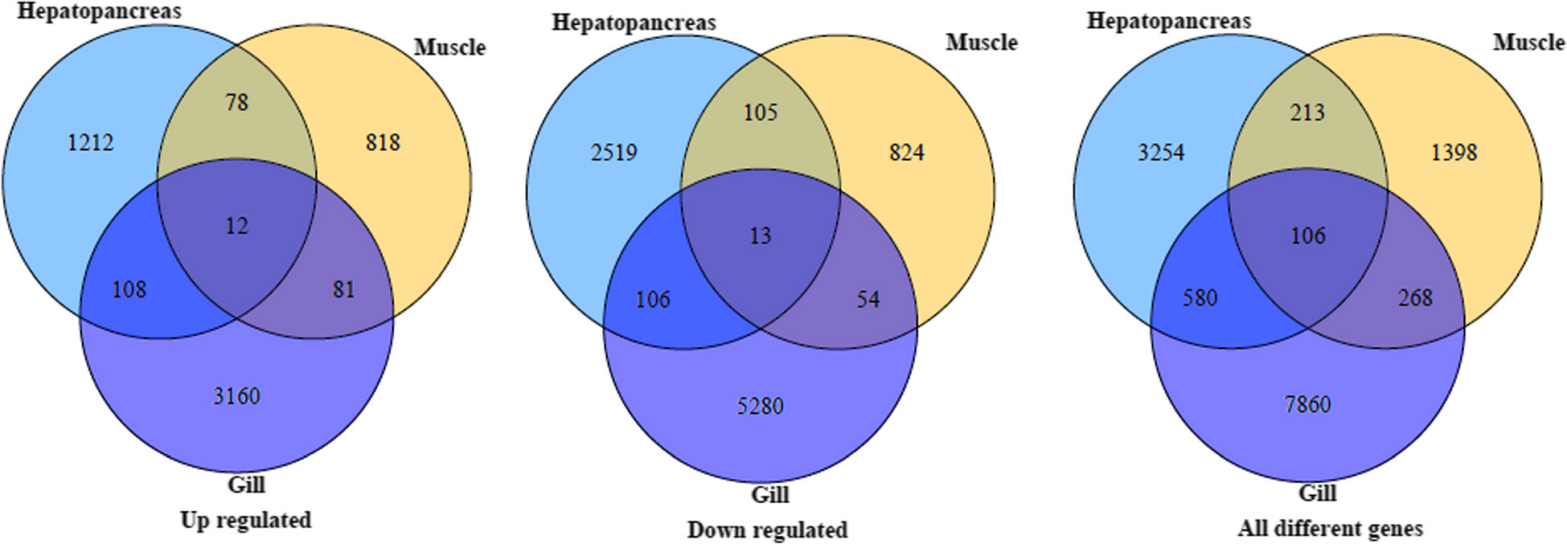
Venn diagram of DEGs among three tissues from *Macrobrachium nipponense*

We detected 13,466 DEGs between hypoxia and normoxia conditions in the hepatopancreas, muscle and gill tissues (false discovery rate ≤0.01 and fold-change ≥2; Fig. 2). Scatter plots were generated for FPKM values from the two treatment groups (Fig. 3). Of these DEGs, 4153, 1985 and 8814 genes were expressed in response to normoxia in hepatopancreas, muscle and gill tissues respectively (Additional file 1A-C). A Venn diagram of the DEGs illustrates that the majority of these genes were not shared amongst the three tissue types, suggesting that the mechanisms and pathways employed in response to chronic hypoxia stress differ significantly between hepatopancreas, gill and muscle tissues (Fig. 4). A key regulator of cellular adaptations to hypoxia is the transcriptional activator hypoxia inducible factor 1 (HIF-1) [30]; however, in our present study with *M. nipponense* – and in previous studies on other invertebrates –HIF-1α mRNA levels in the hepatopancreas consistently showed no response to hypoxia or anoxia [20, 31–34]. The results suggest a model of hypoxia-induced regulation of HIF-1α transcript abundance in crustaceans that may differs from that of typical HIF-1α transcriptional regulation in mammalian systems. Since HIF exerts its action at the level of protein and not mRNA expression level, the absence of HIF among the DEG is not surprising.

**Fig. 5:**
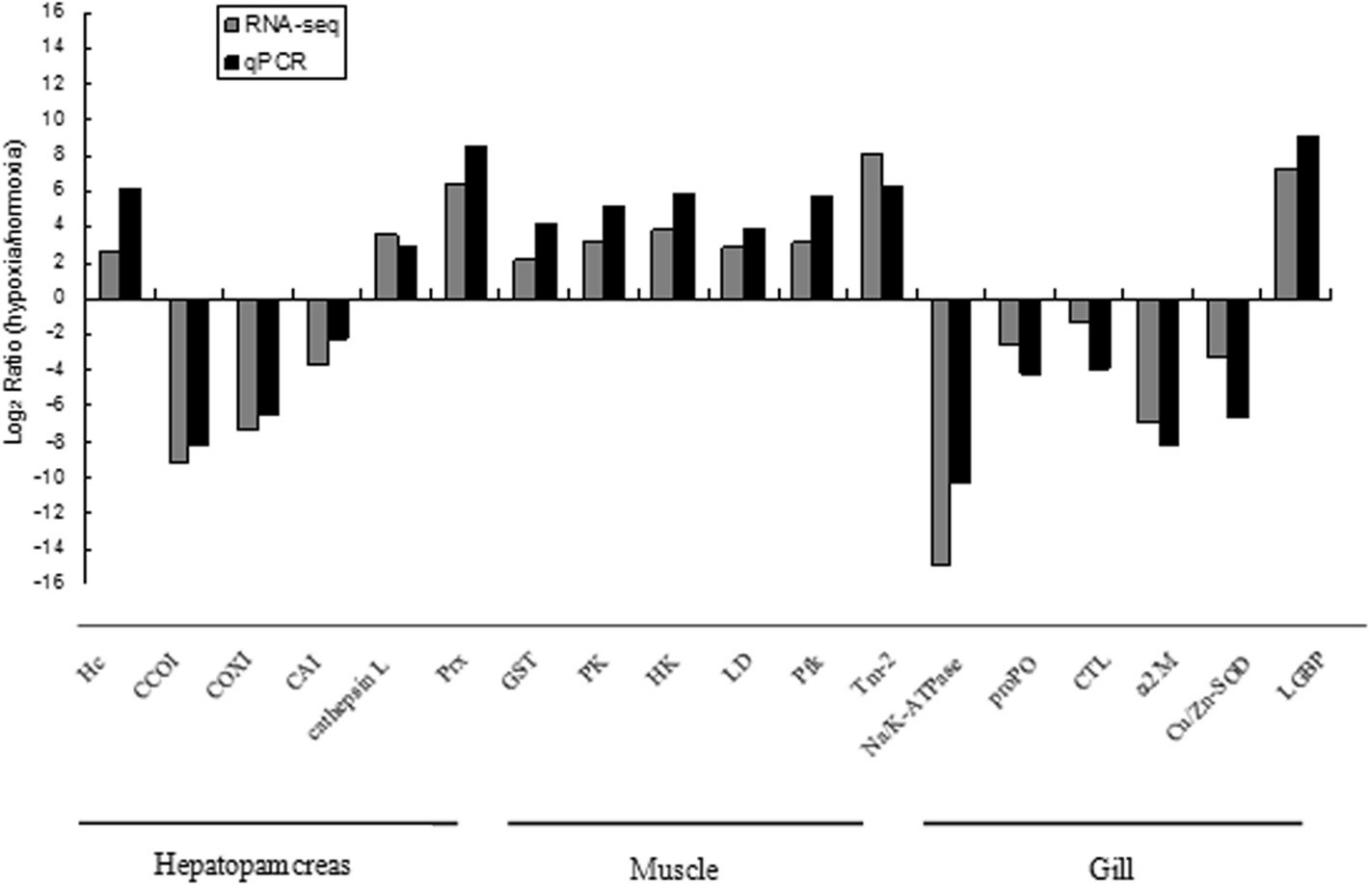
Differentially expressed genes validated by qRT-PCR. Comparison between RNA-Seq results and qRT-PCR validation results. The x-axis shows genes in the three tissues validated in this study; the y-axis shows Log_2_Ratio of expression in *Macrobrachium nipponense* in response to hypoxia versus normoxia conditions

### Functional analysis of DEGs in the hepatopancreas

The metabolic oxygen demands of the hepatopancreas tissues in crustaceans are relatively high and similar across different species [35]. In response to chronic hypoxia, we observed significant gene transcription changes in the hepatopancreas under several GO terms, including upregulated genes for “oxidoreductase activity (GO:0016491)”, “oxidation-reduction process (GO:0055114)” and “catalytic activity (GO:0003824)” and down-regulated genes “electron carrier activity (GO:0009055)”, “immune system process (GO:0002376)” and “ion binding (GO:0043167)” (Table 3). The genes in the molecular function category of “oxidoreductase activity” and “electron carrier activity” are widely studied and known to be associated with oxidative stress, mitochondrial function and adaptation to environmental stress. Moreover, expression profiling studies on other crustaceans have shown that a cluster of genes involved with oxidoreductase activity are differentially expressed in response to environmental stresses, such as temperature and confinement [21, 22, 36, 37]. Genes in the category “electron carrier activity” are responsible for extracting energy, and the slowing down of the respiratory chain caused by a lower oxygen concentration is consistent with reports on stress adaptation and resistance observed for many organisms. For instance, amino acid and ion transmembrane transporters are essential factors for hypoxia responses in many plants [38] and aquatic animals, including mollusks [39] and teleosts [40]. At same time, the mitochondrial electron transport chain is a major site of reactive oxygen species (ROS) production and hypoxic stress could increase hemocyte ROS production, probably because it partially inhibits mitochondrial electron transport producing redox changes in the electron carriers that increase ROS production at complex III [41, 42]. The delicate balance between antioxidant defense and ROS production can be disrupted by compromised antioxidant defense or inhibition of electron flow [43], thus leaving *M. nipponense* potentially vulnerable to ROS damage during hypoxia.

### Functional analysis on DEGs in muscle tissue

In response to chronic hypoxia, in muscle tissues we observed significant enrichment of genes for several GO terms related to stress responses. These GO terms included “catalytic activity (GO:0003824)” and “transcription regulator activity (GO:0030528)” in upregulated genes, and “chitin metabolic process (GO:0006030)” in the down-regulated genes. Since muscle is the main tissue of protein deposition and energy storage, we identified four glycolytic enzyme DEGs in *M. nipponense* in response to hypoxia, suggesting that these were important regulators in response to hypoxia stress in muscles. Under such hypoxic circumstances, the major source of energy is the glycolytic pathway with pyruvate as the substrate, while fermentative metabolism produces lactate via lactate dehydrogenase (LD) [44]. Similar to previous studies [45–47], the primary effects of hypoxia on skeletal muscle were strong increases in the transcription of hexokinase (HK), pyruvate kinase (PK) and LD, which may be a precursor for increased glycolytic ATP production. However, lactate is toxic for the cells and leads to arrest of cellular functions and cell death during hypoxia. Besides its effect on cellular ATP, hypoxia can also influence regulation of the cytoskeleton as reflected by changes in tubulin and myosin proteins. Regulation of myosin by hypoxia has also been shown in the heart muscle of rats [48]. Tubulin-α and -β form the microtubules that are responsible for cellular functions including mitosis, cell motility and shape [49, 50].

### Functional analysis on DEGs in gill tissue

Crustacean gills make an important contribution to immune defenses and have well established functions in gaseous exchange and ion regulation. In response to chronic hypoxia, in gill tissues we observed significant gene enrichment of several GO terms related to stress responses. These GO terms included “oxidoreductase activity (GO:0016901)” and “cell communication (GO:0007154)” in the upregulated genes, and “response to stimulus (GO:0050896)” and “response to chemical stimulus (GO:0042221)” in the down-regulated genes. Notably, there were 15 DEGs in the category “response to stimulus”. We further investigated these DEGs and observed that heat shock protein (HSP) and glutathione S-transferase (GST) genes were down-regulated in the gills in response to hypoxia. HSPs target damaged proteins to the proteasome, which prevents the accumulation of dysfunctional proteins and permits the recycling of peptides and amino acids. This result suggests a high level of autophagy in the gills during hypoxia stress. One of the well-studied transferase families is the GST gene family, as these genes perform essential functions in protecting cells against oxidative stress caused by various stresses, including toxic heavy metal ions [51–53], osmotic imbalance [54], salinity [55, 56] and pH changes [57]. GSTs have been used as biomarkers for environmental pollution and toxin detection [58, 59]. We identified five DEGs belonging to the subcategory “immune system process” and there was down-regulation of prophenoloxidase (proPO), C-type lectin (CTL), antimicrobial peptide (AP) and alpha -2-macroglobulins (α2 M), which are all important for disease resistance in crustaceans [60–63]. Therefore, prolonged hypoxia may have population consequences, as individuals that have already down-regulated their aerobic metabolism may also have decreased immune defenses, which could result in higher risk of outbreaking disease of prawn population.

### Confirmation of DEG candidate genes by real-time reverse-transcriptase polymerase chain reaction (qRT-PCR)

To validate the RNA-Seq results, 18 genes showing a high level of significance or known to play an important role in stress response functions were selected for qRT-PCR analysis with β-actin acting as the reference gene (Table 4). No significant differences were shown between qRT-PCR and the Illumina data (Pearson’s correlation coefficient r = 0.94) (Fig. 5). In addition, a number of detoxification-related genes, such as GST and HSP, were also found to be specifically expressed in different tissues of *M. nipponense* in response to chronic hypoxia. These genes have been shown to be hypoxia-responsive in other studies [12, 21, 22], and they are involved in antioxidant abilities, immune responses, glycolysis and apoptosis, which are important functions for maintaining and re-establishing homeostasis in response to pathological changes. We also found that many genes related to mitochondrial respiration were significantly down-regulated under hypoxia stress according to qRT-PCR, such as cytochrome c oxidase subunit I (CCO I), cytochrome oxidase I (COX I), NADH dehydrogenase subunit 1 (Complex I) and carbonic anhydrase I (CA I), which reveals that normal mitochondrial function was disrupted by oxidative stress. As mitochondrial respiration is affected in many pathologic conditions such as hypoxia and intoxications, the impaired electron transport chain could emit additional p53-inducing signals and thereby contribute to tissue damage [64]. We identified glycolysis related-genes from the DEG list for the muscle, and proPO, CTL, β-1,3-glucan binding protein (LGBP) and α2 M genes from the DEG list for the gill. Obviously, several genes could potentially be used as molecular indicators of hypoxia in *M. nipponense* at specific time points. However, the changes in the expression of these significant genes were too dynamic to serve as biomarkers of hypoxia stress in *M. nipponense*.

### KEGG pathway enrichment analysis

Enrichment analysis is an effective way to identify the KEGG pathways that frequently occur in a tissue when the whole body transcriptome is used as the background [65, 66]. Additional pathway mapping can facilitate the interpretation of significant gene data derived from complex biological processes and systems, especially when trying to characterize DEGs related to environmental toxicants or stressors [67]. Of the significantly expressed genes, 32 % had a match in KEGG pathways targeted by chronic hypoxia. The most abundant categories were associated with oxidative phosphorylation, the MAPK signaling pathway, glycolysis/gluconeogenesis and citrate (TCA) cycle (Table 5). The major biological processes occurring in the mitochondria (TCA cycle, coupling electron transfer and oxidative phosphorylation) were altered remarkably in the hepatopancreas, muscle and gill tissues of *M. nipponense*. These results are in agreement with studies that have reported higher activities of glycolytic enzymes and fatty acid beta-oxidation process after exposure to an acute stressor in fish [68, 69], this may be necessary to cope with the increased energy demand of the tissues. The concentration of mitochondrial oxidative phosphorylation is tuned to the maximum energy conversion requirements of a given tissue, and it is hypothesized that its activity is modulated by tissue metabolic stress to maintain energy-metabolism homeostasis [70].

### Histological studies of the hepatopancreas and gills of *M. nipponense* in response to hypoxia

**Fig. 6:**
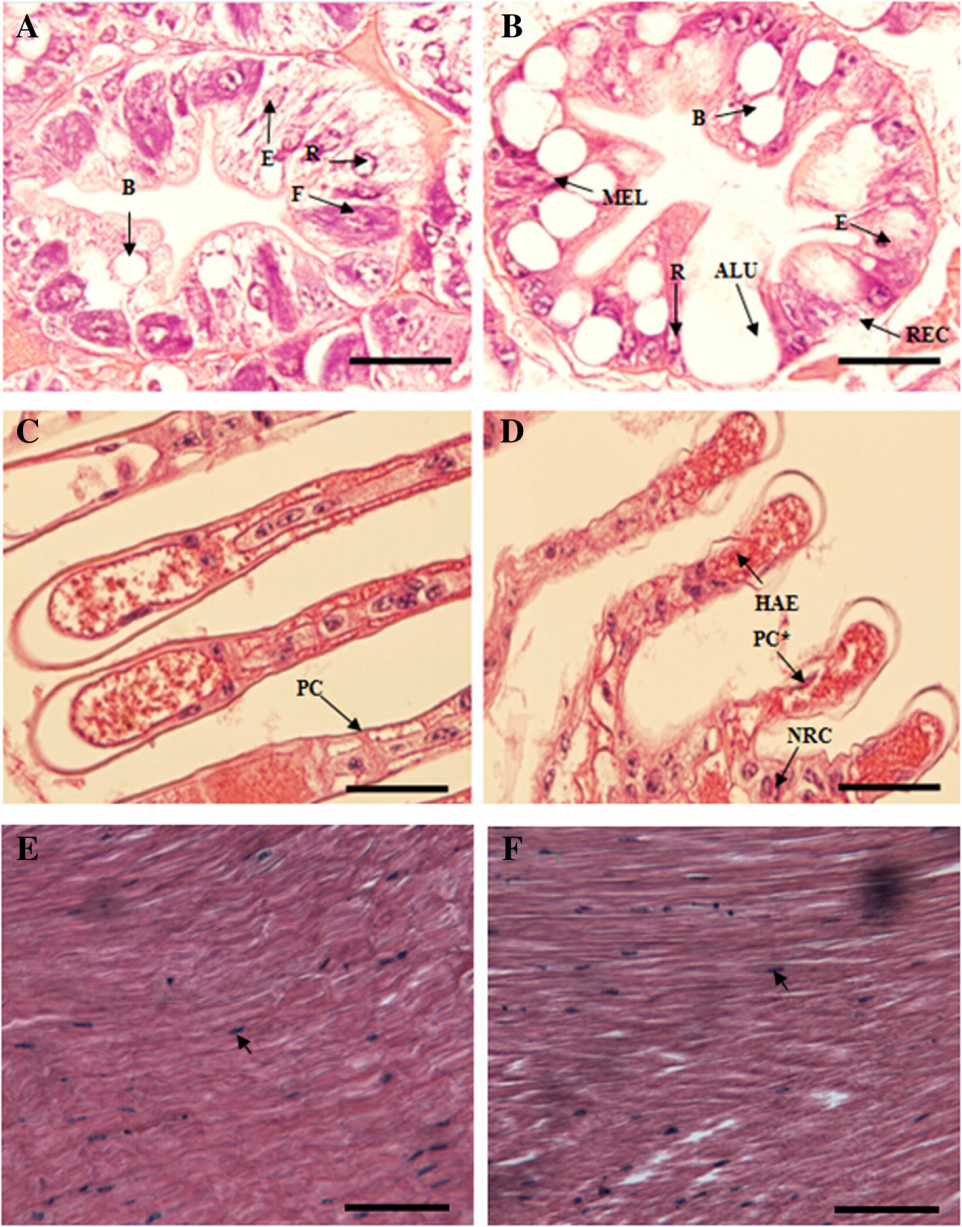
Histopathological changes in the hepatopancreas, gill and muscle. Hepatopancreas of the *Macrobrachium nipponense* following a 7-day exposure to hypoxia: (**a**) control prawns; (**b**) prawns exposed to hypoxia at 2.5 mg/L dissolved oxygen, showing the appearance of large numbers of vacuoles in the tubular epithelial cells and abnormal lumens. ALU, abnormal lumen; MEL, melanization of cells; REC, ruptured epithelial cells. Anterior gill lamellae of the *M. nipponense* following a 7-day exposure to hypoxia: (**c**) control prawns showing normal lamellae structure and intact pillar cells (PC); (**d**) prawns exposed to hypoxia at 2.5 mg/L dissolved oxygen showing disrupted pillar cells (PC*), necrosis (NCR) and extensive infiltration of haemocytes (HAE). Histological cross sections of muscle of (**e**) control group and (**f**) prawns exposed to hypoxia at 2.5 mg/L dissolved oxygen showing simialr striated muscle fibers infiltrated by hemocytes (small arrowhead). Scale bars = 50 μm

The hepatopancreas is composed of numerous blind-end tubules, with each tubule consisting of different epithelial cell types, i.e. E-cell (embryonic), R-cell (resorptive), F-cell (fibrillar) and B-cell (blisterlike) [71]. Histological analysis of the hepatopancreas has been used as a practical means for assessing the environmental stress in the shrimp culture [72–74]. The R-cells in hepatopancreas epithelia are known to function as the main site for lipid storage [74]. There were distinct differences in the condition of the hepatopancreas in *M. nipponense* from normoxia and hypoxia groups (Fig. 6a & b). The most obvious abnormalities were the hypertrophy of B-cells, and vacuoles that tended to coalesce into larger ones. Also, the number of B-cells was increased under hypoxia. The R-cells were compressed, appeared cuboidal and were reduced in number. The tubule lumens were mis-shaped and were enlarged due to a thinned epithelium. In crustaceans, the hepatopancreas is used for monitoring culture health and it serves as a sensitive indicator for metabolism, nutritional status and diseases in various shrimp species because it is the site of digestion, nutrient absorption, reserve storage, and synthesis and secretion of digestive enzymes [75, 76]. Our study has also captured indirect evidence that oxidative stress suppressed antioxidant gene expression and disrupted fatty acid metabolism due to the observation of vacuolation in R-cells. The histoarchitecture of gills in the hypoxia group showed hemocytic infiltration, fused gill lamellae, mild malformations, swollen and necrotic lamellae, and complete disorganization of lamellae (Fig. 6c & d). All these incidents can eventually lead to gill injury, which leads to disturbance in oxygen consumption [77] and enzymatic activities [78] in aquatic organisms. However, no obvious alterations were observed by light microscopy in the muscle tissues of *M. nipponense* after chronic hypoxia (Fig. 6e & f).

## Conclusions

We performed comparative transcriptome profiling of *M. nipponense* in response to hypoxia, and identified a relatively large number of genes that displayed distinct differences in expression in hepatopancreas, muscle and gill tissues. Further analysis revealed that several well-known functional categories of genes and signaling pathways associated with stress responses and energy metabolism were significantly enriched, including genes in the functional categories “response to stimulus”, “transferase activity” and “oxidoreductase activity”, and signaling pathways of “oxidative phosphorylation”, “glycolysis/gluconeogenesis” and “TCA cycle”. Overall, our transcriptome map may provide significant insights into the underlying molecular mechanisms that govern the responses of *M. nipponense* to hypoxia stress. The hypoxia-induced and -reduced genes identified may prove to be potential candidates for global genetic engineering of salt tolerance in *M. nipponense*. The pathways, the gene expression patterns and histological variations in gills and hepatopancreas tissues provide insight into understanding the molecular mechanisms of hypoxia in crustaceans.

## Methods

### Ethics statement

This study was approved by the Animal Care and Use Committee of the Centre for Applied Aquatic Genomics at Chinese Academy of Fishery Sciences.

### Prawn sampling

Several healthy oriental river prawns with wet weights of 2.86–3.45 g were obtained from Tai Lake in Wuxi, China (120°13'44"E, 31°28'22"N). All the samples were transferred to the laboratory of the Freshwater Fisheries Research Center and maintained in six 300-L tanks with aerated freshwater for one week prior to experimentation. During acclimation periods, prawns were hand-fed a commercial feed (Tongwei Group Co., Ltd.) containing 41.0 % crude protein at 5 % wet body weight twice daily. The culture conditions were 22.6 ± 0.5 °C, pH 8.2 ± 0.08, dissolved oxygen 6.5 ± 0.2 mg/L, total ammonia-nitrogen 0.08-0.09 mg/L, and a photoperiod of 14 h light/10 h dark.

The control group was maintained under normoxic conditions (6.5 ± 0.2 mg O_2_/L). Hypoxic (2.5 ± 0.1 mg/L dissolved oxygen) conditions in the treatment tanks were maintained for 7 days by bubbling with N_2_ gas until the desired O_2_ concentrations were reached; oxygen levels were maintained by adding N_2_ gas when needed. Oxygen concentrations were measured daily using a YSI 55oxygen meter (Yellow Springs, OH, USA). All exposures were conducted in triplicate for control and experimental group with three tanks in each group. After 7 days, the hepatopancreas, muscle and gill tissues of six prawns under hypoxia were removed as pooling samples in each tank, respectively. In parallel, six prawns held in air-saturated water (normoxia) were pooled as each negative control tank. Finally, these samples were immediately frozen in liquid nitrogen and stored at -80 °C until being processed. The study was approved by the Institutional Animal Care and Use Ethics Committee of Freshwater Fisheries Research Center, Chinese Academy of Fishery Sciences (Wuxi, China).

### RNA extraction and quality control

Total RNA was extracted from each tissue using TRIZOL (Invitrogen, Carlsbad, CA, USA) according to the manufacturer’s instructions. RNA samples were digested by DNase I to eliminate potential genomic DNA. Integrity and size distribution were checked on a Bioanalyzer 2100 with RNA 6000 Nano Labchips (Agilent technologies, Santa Clara, CA, USA). Equal amounts of the high quality RNA sample from each tissue were then pooled for RNA-Seq. cDNA library construction and sequencing and RNA-Seq library preparation and sequencing were carried out as previously described [79]. cDNA libraries were prepared with 2.5 μg total RNA following the protocols of the Illumina TruSeq RNA Sample Preparation Kit (Illumina). After KAPA quantitation and dilution, the library was sequenced on an Illumina HiSeq 2000 with 100-bp paired-end reads. After removing adaptor sequences, ambiguous ‘N’ nucleotides (with the ratio of ‘N’ greater than 5 %) and low quality sequences (with quality score less than 10), the remaining clean reads were assembled using trinity software [29] as described for de novo transcriptome assembly without a reference genome and this generated the reference sequences including a number of EST sequences available in public domains for the comparative transcriptome study.

### Functional annotation of assembled contigs

The assembled transcriptome contigs were subjected to similarity searches against the NCBI non-redundant (nr) protein database using BLASTx with e-value cutoff of 1e-10. Gene name and description was assigned to each contig based on the BLASTx hit with the highest score. GO analysis was conducted on the assembled transcriptome using InterProScan (http://www.ebi.ac.uk/Tools/pfa/iprscan/) and integrated protein databases with default parameters. The GO terms associated with transcriptome contigs were then obtained for describing their biological processes, molecular functions and cellular components. For pathway enrichment analysis, all DEGs were mapped to terms in the KEGG database and searched for significantly enriched KEGG terms compared to the whole transcriptome background. Functional enrichment analysis, including GO and KEGG, were performed using the ultra-geometric test to identify which DEGs were significantly enriched in GO terms (P-value ≤0.05) and metabolic pathways (q-value ≤0.05) compared with the whole transcriptome background [80, 81].

### Read mapping and DEG analysis

All the cleaned reads were mapped to the assembled reference transcriptome by Bowtie [28], and about 70 % of the reads could be mapped to the reference for each sample (Table 1). RSEM was then used to estimate and quantify gene and isoform abundances according to the Trinity-assembled transcriptome. Finally, we used edgeR to normalize the expression levels in each of these samples and obtain the differentially expressed transcripts by pairwise comparisons [82]. EdgeR uses a negative binomial distribution method for differential expression analysis.

### qRT-PCR

qRT-PCR was used to validate the expression of 18 DEGs. The sequences of the primer pairs (designed using Primer Express 3.0) are listed in Additional file 2. qRT-PCR reactions were carried out using the Bio-Rad iCycler iQ5 Real Time System (Biorad Inc., Berkeley, CA, USA) using the β-actin gene as internal control [83]. The PCR temperature profile and reaction conditions were performed according to the instructions of the SYBR Premix Ex Taq kit (TaKaRa, Dalian, China). For the negative control, diethypyrocarbonate (DEPC)-water replaced the template. A relative standard curve was constructed using 10-fold serially diluted cDNA. Each sample was run in triplicate along with the internal control gene. To ensure that only one PCR product was amplified and detected, a dissociation curve analysis of amplification products was performed at the end of each PCR reaction. The relative copy number of gene mRNAs was calculated according to the 2^-∆∆CT^ comparative CT method [84]. Pearson correlations between the qPCR data (average expression for each experimental group) and the Illumina data obtained by RNA-seq were calculated for each candidate gene.

### Histopathology

Due to the hepatopancreas, gill and muscle of prawns being the main organs where multiple oxidative reactions, respiratory metabolism and genergy production occur with high metabolic activity. Three prawns from the hypoxia and normoxia groups were used to determine the histopathological effects of 7 days of hypoxia on the hepatopancreas, gill and muscle of prawns. The hepatopancreas, gill and muscle were removed and preserved in 10 % neutral-buffered formalin for 7 days. Then the samples were rinsed in 70 % ethanol and stored until further processing. The samples were dehydrated in isopropanol, cleared in xylene, infiltrated in paraffin, and then sectioned to a thickness of 5 μm. Sections were stained with hematoxylin and eosin and examined with a light microscope (Nikon Phase Contrast 0.90 Dry Japan). Hepatopancreas, gill and muscle tissue morphology was assessed in order to observe possible alterations revealed through differences in the condition of cell morphology.

### Availability of supporting data

Raw sequencing data is available through the NCBI Sequence Read Archive under Project Accession SRP056408 (http://www.ncbi.nlm.nih.gov/sra/?term=SRP056408).

## Additional files

Additional file 1: DGE in three different tissues. Additional file 2: Primers used in validation of DEGs.
