# Supplementary material for: Transciptomic and histological analysis of hepatopancreas, muscle and gill tissues of oriental river prawn (Macrobrachium nipponense) in response to chronic hypoxia
Source: BMC Genomics. 2015 Jul 3;16(1):491. doi: 10.1186/s12864-015-1701-3 (PMC4490754; doi:10.1186/s12864-015-1701-3)
Supplement: Additional file 2: — Primers used in validation of DEGs. [file 12864_2015_1701_MOESM2_ESM.doc]

Table A.1. Primers used in the present study

| Target mRNA | Sequence (5′-3′) |
| --- | --- |
| hemocyanin-F | CGCGGATCCTCCACTAGTGATTTC |
| hemocyanin-R | CTGAAGGGAACATACGCATAAGG |
| cytochrome c oxidase subunit I-F | CGCCCACGCTTTCGTAATAAT |
| cytochrome c oxidase subunit I-R | GGGAATGCTATATCTGGCGCT |
| cytochrome oxidase subunit I-F | TATTAGGAGCGCCAGACATAGC |
| cytochrome oxidase subunit I-R | GGGGTAGACAGTTCATCCTGTG |
| carbonic anhydrase I-F | GTTCCACTTCCACTGGGGAG |
| carbonic anhydrase I-R | GCTTCCGTAAGAGCCCTTGT |
| cathepsin L-F | GTGGTAATGGAGGAGTGGGAG |
| cathepsin L-R | TAGAGCTTGTTGTGGGCAGC |
| Prx-F | CTTGGATCAACACCCCACGTA |
| Prx-F | CCTCTGAAGGCGATACCATCC |
| glutathione S-transferase-F | GCCCCAAAGTTTGACAAGTCT |
| glutathione S-transferase-R | AATGGCATTGCTCTGGGTTAC |
| pyruvate kinase-F | CCAAATGTCGCCCGTCAATG |
| pyruvate kinase-R | TCTGCCATCCAGTGACAACC |
| hexokinase-F | TGTTCCCCAGCCGATTATGG |
| hexokinase-R | CGGCGCACTTGAATCCTTTG |
| lactate dehydrogenase-F | TGACAGACGCCGAGAAGAAC |
| lactate dehydrogenase-R | GGCAACTGGATGTCGAAGGT |
| 6-phosphofructokinase-F | ATCCATGGGTCAGCGTTTGA |
| 6-phosphofructokinase-R | CCGAGTACGGTAATGCGTGT |
| Na/K-ATPase-F | CAGAGCAGATGGTGGATGTTG |
| Na/K-ATPase-R | ATGTACCAGAGGGTCTGTTGG |
| prophenoloxidase-F | CGTGACCGTCCGAATTTTCTT |
| prophenoloxidase-R | GACGATCTCACCAGCTTGTTC |
| tropomyosin-2-F | GCAGAGAGATCTGTGCAGAAGC |
| tropomyosin-2-R | GTAGCCAGACAGTTCGCTGAAT |
| C-type lectin-F | CGGACATTGTGAAACCAGTGT |
| C-type lectin-R | CCAACTCGTTGGCATCATTCA |
| α-Macroglobulins-F | CTTGGGTTACTTTCGTCGGTC |
| α-Macroglobulins-R | TCGGTGGAAGTCGAAATCGTA |
| Cu/Zn-SOD-F | GGGCAGGCTTTACGGTGCTCTT |
| Cu/Zn-SOD-R | GGGCAGGCTTTACGGTGCTCTT |
| β-1,3-glucan binding protein-F | GTCACGATGGCAAGCTCTTC |
| β-1,3-glucan binding protein-R | CAGGCCAAGTACCGTAAGCA |
| *β*-actinF | AATGTGTGACGACGAAGTAG |
| *β*-actinR | GCCTCATCACCGACATAA |
